# DualPose: Dual-Block Transformer Decoder with Contrastive Denoising for Multi-Person Pose Estimation

**DOI:** 10.3390/s25102997

**Published:** 2025-05-09

**Authors:** Matteo Fincato, Roberto Vezzani

**Affiliations:** Department of Engineering “Enzo Ferrari”, University of Modena and Reggio Emilia, Via P. Vivarelli 10, 41125 Modena, Italy; matteo.fincato@unimore.it

**Keywords:** contrastive denoising, DualPose, human pose estimation, multi-person pose estimation, transformer-based models

## Abstract

Multi-person pose estimation is the task of detecting and regressing the keypoint coordinates of multiple people in a single image. Significant progress has been achieved in recent years, especially with the introduction of transformer-based end-to-end methods. In this paper, we present DualPose, a novel framework that enhances multi-person pose estimation by leveraging a dual-block transformer decoding architecture. Class prediction and keypoint estimation are split into parallel blocks so each sub-task can be separately improved and the risk of interference is reduced. This architecture improves the precision of keypoint localization and the model’s capacity to accurately classify individuals. To improve model performance, the Keypoint-Block uses parallel processing of self-attentions, providing a novel strategy that improves keypoint localization accuracy and precision. Additionally, DualPose incorporates a contrastive denoising (CDN) mechanism, leveraging positive and negative samples to stabilize training and improve robustness. Thanks to CDN, a variety of training samples are created by introducing controlled noise into the ground truth, improving the model’s ability to discern between valid and incorrect keypoints. DualPose achieves state-of-the-art results outperforming recent end-to-end methods, as shown by extensive experiments on the MS COCO and CrowdPose datasets. The code and pretrained models are publicly available.

## 1. Introduction

The task of detecting and regressing the keypoint positions of multiple individuals on data coming from camera sensors is known as multi-person pose estimation. Considerable progress has been made in multi-person pose estimation over the last few years. Methods are generally divided into two categories: non-end-to-end and end-to-end. Top-down [1,2,3,4,5,6], bottom-up [7,8,9,10], and one-stage model [11,12,13,14] approaches are examples of non-end-to-end methods.

Top-down methods, which are based on a highly accurate person detector, identify people in an image first and then estimate their poses. Bottom-up methods are effective in crowded environments, as they predict all keypoints in the image and then associate them into individual poses, thus removing the need for explicit detection. One-stage methods predict keypoints and their associations directly, simplifying the process by combining pose estimation and detection into a single step.

Transformers were originally introduced by Vaswani et al. [15] for machine translation. One of the main advantages of transformers is their global computations and perfect memory, which makes them more suitable than RNNs for long sequences. Using this ability to handle sequences, DETR [16] modeled the object detection problem as generating a sequence of rectangles, one for each identified object. The same DETR paradigm was then extended to pose estimation, where the four-dimensional representation of the detection box becomes a sequence of joint positions. PRTR [17] and TFPose [18] are two seminal examples to which the reader can refer for further details. Inspired by the success of DETR, end-to-end methods use transformers to improve accuracy and performance by integrating the entire process into a single framework.

By treating object detection as a direct prediction of a set of objects, these techniques transform multi-person pose estimation and eliminate the need for post-processing steps such as non-maximum suppression. GroupPose [19] optimizes the pose estimation pipeline and achieves superior performance by using group-specific attention mechanisms to refine interactions between keypoint and instance queries.

**Figure 1:**
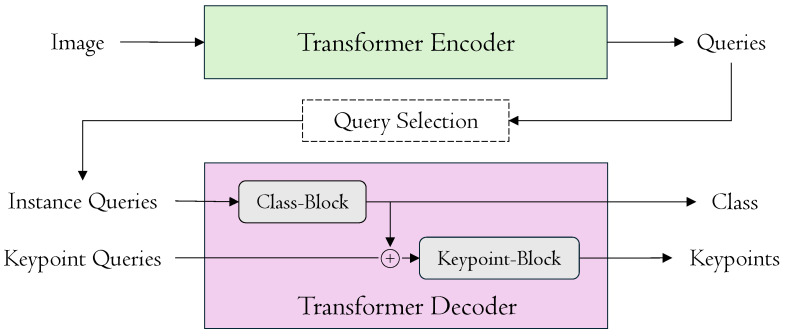
DualPose scheme. DualPose operates through a two-stage strategy, where an image coming from the camera sensor is processed by an encoder to generate queries. These queries are then filtered and passed into a decoder, which is composed of two blocks: the Class-Block, responsible for class predictions, and the Keypoint-Block, which handles keypoint localization. The final outputs include both class predictions and keypoints coordinates.

We build on this line of work and introduce a new and effective framework for end-to-end multi-person pose estimation called DualPose, as depicted in Figure 1. DualPose presents significant innovations that improve computational effectiveness and accuracy. First, we modify the transformer decoder architecture by employing two distinct blocks: one for processing class prediction and the other for keypoint localization. Inspired by GroupPose [19], we use N×K keypoint queries to regress N×K positions and *N* class queries to categorize human poses. For class queries, the Class-Block transformer accurately identifies and categorizes the people in the input image. The output of this Class-Block transformer is then added to the Keypoint-Block transformer, providing more comprehensive contextual information. Furthermore, in comparison to conventional sequential processing, we introduce an improved strategy within the Keypoint-Block by employing a novel parallel computation technique for self-attentions to enhance both efficiency and accuracy.

To stabilize training and improve effectiveness, DualPose also includes a contrastive denoising (CDN) mechanism [20]. This contrastive method improves the model’s ability to differentiate real and false keypoints. We use two hyperparameters to control the noise scale, generating positive samples with lower noise levels (to reconstruct their corresponding ground-truth keypoint positions) and negative samples with heavily distorted poses, which the model learns to predict as “no pose”. Both types of samples are generated based on ground-truth data. Furthermore, we present a novel adaptation, inherited from object detection [20], by combining both L1 and object keypoint similarity (OKS) as reconstruction losses. The L1 loss ensures fine-grained accuracy in keypoint localization, while the OKS loss accounts for variations in human pose structure and aligns the predicted keypoints with ground-truth annotations.

Experimental results demonstrate that our novel approach, DualPose, outperforms recent end-to-end methods on both MS COCO [21] and CrowdPose [22] datasets. Specifically, DualPose achieves an AP of 71.2 with ResNet-50 [5] and 73.4 with Swin-Large [23] on the MS COCO val2017 dataset.

In this work, we introduce key innovations that improve the accuracy and effectiveness of multi-person pose estimation: nolistsep

- DualPose uses a dual-block decoder to separate class prediction from keypoint localization, reducing task interference.
- Parallel self-attention in the keypoint block improves effectiveness and precision.
- A contrastive denoising mechanism enhances robustness by helping the model distinguish between real and noisy keypoints.
- L1 and OKS losses are applied exclusively for keypoint reconstruction, rather than for bounding boxes as in object detection.

## 2. Related Work

Multi-person pose estimation has seen substantial advances, with methodologies commonly divided into non-end-to-end and end-to-end approaches. Non-end-to-end methods include top-down and bottom-up strategies, as well as one-stage models. End-to-end methods integrate the entire process into a single streamlined framework.

### 2.1. Non-End-to-End Methods

Non-end-to-end approaches for multi-person pose estimation encompass both top-down [1,2,3,4,6,24] and bottom-up [7,8,9,10] methods.

Top-down methods identify individual subjects in an image and then estimate their poses. However, the accuracy of person detection is a major factor in how effectively they perform especially in busy or obscure environments. The two-step procedure also amplifies the risk of error propagation and increases processing complexity.

Bottom-up methods detect all keypoints first and then associate them to form poses. This approach is more efficient in crowded scenes, but adds complexity during keypoint association, often resulting in reduced accuracy in challenging scenarios.

We also include in this section the recent methods that make use of diffusion models, such as DiffusionRegPose [25], *Di*^2^*Pose* [26]: the best performances are in fact obtained by exploiting their iterative capacity for continuous improvement of the result. Therefore, they are not able to produce the final results in a single step. Moreover, they require an initial (random) seed posture as a first step for each detected person, falling in the top-down category. However, we believe they are of great importance, especially for their ability to generate multiple hypotheses, thus exploiting all the potential of probabilistic methods.

One-stage methods [11,12,13,14,27] streamline pose estimation by combining detection and pose estimation into a single step. These models directly predict keypoints and their associations from the input image, eliminating intermediate stages such as person detection or keypoint grouping.

### 2.2. End-to-End Methods

End-to-end methods [19,28,29,30] revolutionize multi-person pose estimation by integrating the entire pipeline into a single, unified model. Inspired by detection transformer (DETR) [16], which pioneered the use of transformers for object detection, along with its variants [20,31,32,33,34,35], these methods streamline the process and improve accuracy.

PETR [28] employs a transformer-based architecture to solve multi-person pose estimation as a hierarchical set prediction problem, first regressing poses using a pose decoder and then refining keypoints using a keypoint decoder. QueryPose [29] adopts a sparse query-based framework to perform human detection and pose estimation, eliminating the need for dense representations and complex post-processing. ED-Pose [30] integrates explicit keypoint box detection to unify global (human-level) and local (keypoint-level) information, enhancing both efficiency and accuracy through a dual-decoder architecture. GroupPose [19] uses group-specific attention to refine keypoint and instance query interactions, optimizing the process and achieving superior performance with one simple transformer decoder.

These methods have limitations: PETR struggles with keypoint precision and high computational costs, ED-Pose adds complexity with its dual-decoder, and QueryPose falters with occlusions.

GroupPose still employs a single decoder that mixes classification and keypoint regression, causing task interference in crowded scenes, and its within/across attentions are executed sequentially, adding latency and increasing FLOPs compared with our parallel dual-block design.

Our model addresses these issues with a dual-block transformer that separates class and keypoint predictions, enhancing precision and effectiveness, while the contrastive denoising mechanism improves robustness without added complexity.

Finally, we reiterate that DualPose is currently developed to work on traditional RGB images. Architectures that operate on depth sensors [36,37] or on data from radar sensors [38] have not been taken into consideration.

## 3. Proposed Method

We propose DualPose, a novel and effective framework for end-to-end multi-person pose estimation. Building on previous end-to-end frameworks [19,28,29,30], we formulate the multi-person pose estimation task as a set prediction problem. DualPose employs a more advanced dual-block transformer decoder compared to previous approaches, which only use a basic transformer decoder [31]. This improves accuracy and computational efficiency. In the following sections, we detail the core components of DualPose.

### 3.1. Overall

**Figure 2:**
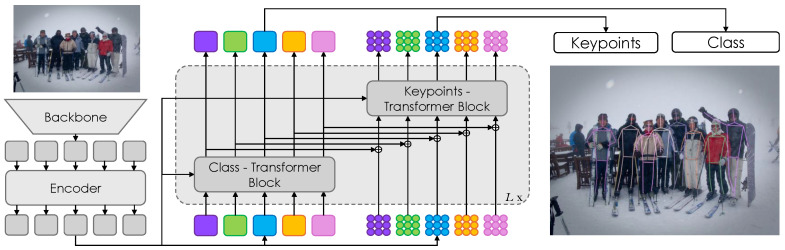
DualPose. Overview of the proposed system: the backbone extracts features from the image given by the sensor and provides them to multiple encoder layers. The output is then given to the decoder (middle), which uses two different transformer blocks: the Keypoint-Transformer Block, which focuses on keypoint prediction, and the Class-Transformer Block, which is dedicated to class prediction. To improve the keypoint predictions, the Class-Transformer Block’s output is merged into the Keypoints-Transformer Block. The final output integrates class and keypoint predictions for each detected human instance.

The DualPose model consists of four primary components: a backbone [5,23], a transformer encoder [15], a transformer decoder, and task-specific prediction heads. This architecture is designed to simultaneously compute *K* keypoints (e.g., K=17 on MS COCO) for *N* human instances in the input image coming from an RGB sensor. Refer to Figure 2 for the complete model architecture.

#### 3.1.1. Backbone and Transformer Encoder

We follow the DETR framework [31] to build the transformer encoder and backbone. The model processes an input image and generates multi-level features using six deformable transformer layers [31]. These features are then sent to the following transformer decoder.

#### 3.1.2. Transformer Decoder

We introduce a dual-query system for the transformer decoder, where *N* instance queries and N×K keypoint queries are generated. The instance queries classify each person in the image, while the keypoint queries predict the positions of the keypoints for each detected individual. By treating each keypoint independently, these keypoint queries allow for precise regression of the N×K keypoint positions. This separation of tasks enables the model to handle instance detection and keypoint localization more effectively.

Each decoder layer follows the design of previous DETR frameworks, incorporating self-attention, cross-attention implemented with deformable attention, and a feed-forward network (FFN). However, each decoder layer architecture has been extended to include two distinct blocks: one for class prediction and one for keypoint prediction. Unlike GroupPose [19], where two group self-attentions are computed sequentially, our method computes them in parallel.

#### 3.1.3. Prediction Heads

Following GroupPose [19], we employ two lightweight three-layer feed-forward heads (hidden width 256) for human classification and key-point regression. The classification head converts each instance query into a soft-max confidence for the human class plus a background label, while the regression head projects the same query embedding to a 2K-dimensional vector containing the normalized (x,y) coordinates of the *K* joints. During inference DualPose outputs, for every one of the *N* instance queries, both its classification score and the full set of *K* keypoint locations; a simple confidence threshold is then applied to discard low-quality poses.

#### 3.1.4. Losses

The loss function in DualPose addresses the assignment of predicted poses to their corresponding ground-truth counterparts using the Hungarian matching algorithm [16], ensuring a one-to-one mapping between predictions and ground-truth poses. It includes classification loss ($L_{\mathrm{cls}}$) and keypoint regression loss ($L_{\mathrm{kpt}}$), without additional supervisions such as those in QueryPose [29] or ED-Pose [30]. The keypoint regression loss ($L_{\mathrm{kpt}}$) combines $\ell_{1}$ loss and object keypoint similarity (OKS) [28]. The cost coefficients and loss weights from ED-Pose [30] are used in Hungarian matching and loss calculation.

The overall loss is then computed as following:

(1) $$L=L_{\mathrm{cls}}+L_{\mathrm{kpt}},$$

(2) $$L_{\mathrm{kpt}}=\;\delta_{1}\sum_{j=1}^{K}{∥{\hat{p}}_{j}-p_{j}∥}_{1}+\;\delta_{2}\;\left(1-\mathrm{OKS}(\hat{p},p)\right)$$

where ${\hat{p}}_{j}$ and $p_{j}$ are the predicted and ground-truth coordinates of joint *j*, and $\delta_{1},\delta_{2}$ are the weights as defined by Yang et al. [30].

### 3.2. Queries

In multi-person pose estimation, the objective is to predict *N* human poses, each with *K* keypoint positions for every input image. To achieve this, we use N×K keypoint queries, where each set of *K* keypoints represents a single pose. Furthermore, *N* instance queries are used to classify and score the predicted poses, ensuring accurate identification and assessment of each human instance.

### 3.3. Query Construction

The process begins by identifying human instances and predicting poses using the output memory of the transformer encoder, following previous frameworks [19,28,30]. We select *N* instances based on classification scores, resulting in N×D memory features.

For keypoint queries, each content part (K×D) is constructed by combining randomly initialized learnable keypoint embeddings with corresponding memory features. The position part is initialized based on the predicted human poses. Instance queries use randomly initialized learnable instance embeddings (1×D) for classification tasks, without explicit position information.

### 3.4. Contrastive Denoising

**Figure 3:**
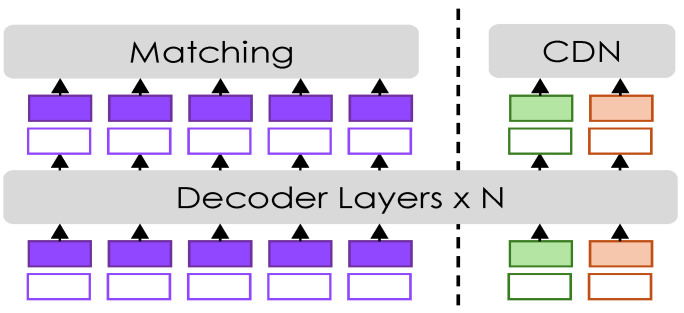
Contrastive denoising training. Additional queries are introduced, where the reference poses are derived from the ground truth (GT) combined with noise. These queries are then divided into positive and negative samples based on the amount of noise added to the GT.

In this work, we improve training stability and performance by integrating a contrastive denoising (CDN) mechanism into the transformer decoder. During training, CDN adds controlled noise to the ground-truth labels and poses, helping the model to better distinguish between true and false keypoints. Our approach is inspired by the work of Zhang [20], which applies CDN to object detection. However, we have adapted it to suit the context of pose estimation, allowing our model to achieve greater robustness in discerning accurate poses from noisy data. A summary schema is shown in Figure 3.

To this end, we use two different hyperparameters, $\lambda_{1}$ and $\lambda_{2}$, to control the noise scale for keypoints in pose estimation. Here, $\lambda_{1}<\lambda_{2}$, ensuring a hierarchy in noise levels. Positive queries are designed to closely reconstruct ground-truth (GT) keypoints and are generated within an inner boundary defined by $\lambda_{1}$. Negative queries are meant to predict “no pose”, simulating difficult scenarios that force the model to distinguish between true and false positives. They are placed between the inner and outer boundaries ($\lambda_{1}$, $\lambda_{2}$).

Let $p_{\mathrm{gt}}\;=\;\left(x_{\mathrm{gt}},y_{\mathrm{gt}}\right)$ be a ground-truth keypoint. We sample ε∼U(−1,1) and generate

(3) $$p^{+}=p_{\mathrm{gt}}+\varepsilon \;\lambda_{1}\;\;p^{-}=p_{\mathrm{gt}}+\lambda_{1}+\varepsilon \;\left(\lambda_{2}-\lambda_{1}\right).$$

Positive and negative queries are organized into CDN groups. If the input image contains *n* ground-truth (GT) poses, each CDN group generates 2×n queries, with each GT pose yielding a positive and a negative query. The reconstruction losses are $L_{1}$ and OKS for keypoint regression, and focal loss for classification. An additional focal loss is used to label negative samples as background.

This contrastive denoising method greatly enhances the model’s performance in multi-person pose estimation by improving its ability to accurately identify keypoints despite varying degrees of noise.

### 3.5. Dual-Block Decoder

In DETR frameworks [16,31,32], the self-attention of the transformer decoder captures interactions between queries, but processing keypoint regression and class predictions simultaneously can lead to inefficiencies and interference between tasks. This often results in reduced accuracy and precision when handling complex scenes with multiple individuals or occlusions.

To address these issues, we propose a new transformer decoder architecture in DualPose that decouples the computation of keypoints and class predictions into two distinct blocks, allowing each one to process the queries independently and more effectively. This division allows a more specialized handling of the corresponding task, with the Class-Block focused on class prediction and the Keypoint-Block on keypoint localization. This dual-block structure minimizes interference between the different query types, improving accuracy and effectiveness in multi-person pose estimation.

#### 3.5.1. Class-Block

The Class-Block transformer processes class queries independently from keypoint-queries for class prediction. As a result, it can focus on accurately identifying individuals across the image. The output of the Class-Block is then added to the input of the Keypoint-Block, providing richer contextual information.

#### 3.5.2. Keypoint-Block

The Keypoint-Block transformer is designed to handle the prediction of body joints. We incorporate the group self-attention mechanism from GroupPose [19], but we introduce a new enhancement by processing these self-attentions in parallel. Specifically, the queries, keys, and values are split into two halves, each processed by a separate self-attention mechanism simultaneously. This design boosts effectiveness and accuracy in predicting keypoints, while leveraging the refined contextual information from the Class-Block.

#### 3.5.3. Parallel Group Self-Attention

Let $Q\;\in \;R^{(N\times K)\times d}$ be the keypoint query matrix (*N* people, *K* joints each, model width *d*). We first split the feature dimension into two equal halves:

(4) $$Q\;=\;\left[\;Q^{\left(1\right)}\;∥\;Q^{\left(2\right)}\right],\;Q^{\left(1\right)},Q^{\left(2\right)}\in R^{\left(N\times K)\times (d/2\right)}.$$

For each sub-matrix we build its own triplet $\left(\;Q^{\left(i\right)},K^{\left(i\right)},V^{\left(i\right)}\right)$ (i=1,2) using the same feature for queries, keys and values, as customary in self-attention: $K^{\left(i\right)}=Q^{\left(i\right)},\;V^{\left(i\right)}=Q^{\left(i\right)}$. We decompose the full self-attention map into two parallel group-attentions:

- Within-group attention: a block of size K×K repeated for every person, so that each *K* keypoint queries for one instance attend only to each other. This captures the kinematic relations inside one human pose.
- Across-group attention: a block of size N×N repeated for every keypoint type. It lets equal-type queries exchange information across different people, enabling duplicate suppression and global context.

Their outputs are concatenated and projected back to the original width:

(5) $$Z=\mathrm{Proj}\;\left[\;Z_{s}\;∥\;Z_{t}\;\right]\;\in \;R^{(N\times K)\times d}.$$

## 4. Experiments

In this section, we describe the experimental setting, including the dataset and the training protocol used in our pipeline, and subsequently compare our results with state-of-the-art approaches.

### 4.1. Settings

#### 4.1.1. Dataset

In our experiments, two popular datasets for human pose estimation are used: MS COCO [21] and CrowdPose [22]. MS COCO contains 250,000 person instances across 200,000 images, each with 17 keypoint annotations (we set K=17 for this dataset). DualPose is evaluated using the COCO val2017 and test-dev sets after being trained on the COCO train2017 set.

CrowdPose includes 20,000 images with 80,000 person instances, each with 14 keypoint annotations (K=14 for CrowdPose). Since crowded and occluded scenes occur frequently, CrowdPose poses additional challenges. We use the standard CrowdPose split 5:1:4 released with the dataset: 10,000 images for training, 2000 for validation, and 8000 for testing.

#### 4.1.2. Evaluation Metrics

The OKS-based average precision (AP) scores serve as the primary evaluation metric for both datasets. For MS COCO, we report AP scores (in percentage) across various thresholds and object sizes (medium and large), specifically denoted as AP, $AP_{50}$, $AP_{75}$, $AP_{M}$, and $AP_{L}$, following the standard evaluation protocol. In the case of CrowdPose, we evaluated model performance under different crowding conditions using AP scores at different thresholds and crowding levels, labeled as AP, $AP_{50}$, $AP_{75}$, $AP_{E}$, $AP_{M}$, and $AP_{H}$, which represent easy, medium, and hard crowding scenarios, respectively.

#### 4.1.3. Implementation Details

Our training and testing procedures follow those of ED-Pose [30] and GroupPose [19]. We utilize the AdamW optimizer [39,40] with a weight decay of $1\times 10^{-4}$. We set the base learning rate to $1\times 10^{-4}$ and the backbone’s learning rate to $1\times 10^{-5}$, consistent with DETR frameworks. A total batch size of 16 is adopted. The model is trained for 60 epochs on MS COCO [21] and 80 epochs on CrowdPose [22]. The learning rate is decayed by a factor of 0.1 at the 50th epoch for MS COCO and at the 70th epoch for CrowdPose. We use common data augmentations, such as random flips, crops, and resizing, as in DETR frameworks [16,20,30,31] during training. Images are resized so that the long side does not exceed 1333 pixels, and the short side is between 480 and 800 pixels. All experiments are conducted on 2 × NVIDIA A40 GPUs.

### 4.2. Experimental Results

Our goal is to develop an effective framework for end-to-end multi-person pose estimation. Hence, we primarily compare DualPose with previous end-to-end frameworks, such as PETR [28], QueryPose [29], ED-Pose [30], and GroupPose [19]. Additionally, to demonstrate the effectiveness of our approach, we include comparisons with non-end-to-end frameworks, encompassing top-down [17,41], bottom-up [10,42,43,44], and one-stage methods [11,13,27,45].

#### 4.2.1. Comparisons with End-to-End Frameworks on COCO

The comparisons on the COCO val2017 set and test-dev set are shown in Table 1 and Table 2. These findings confirm that DualPose performs consistently better than PETR [28] and QueryPose [29], and is on par with ED-Pose [30] and GroupPose [19]. For fairness, every model is a retrained on 2 × NVIDIA A40 GPU.

DualPose, using ResNet-50 [5] as its backbone, achieves an average precision (AP) of 71.2% on the COCO val2017, outperforming models such as PETR (67.4%) and QueryPose (68.0%). DualPose improves further when using the Swin-Large [23] backbone, reaching an AP of 73.4%, comparable to GroupPose (72.8%) and ED-Pose (72.0%), indicating its competitiveness with state-of-the-art models. Furthermore, the values for AP_50_, AP_75_, AP_*M*_, and AP_*L*_ reflect the same trend as the overall AP, highlighting the effectiveness of the dual-block transformer decoder and contrastive denoising mechanism.

On the COCO test-dev set, DualPose achieves 69.5%, 70.8%, and 71.8% AP with ResNet-50 [5], Swin-Tiny [23], and Swin-Large [23] backbones, respectively. These results confirm DualPose’s role as a top-performing framework for multi-person pose estimation.

#### 4.2.2. Comparisons with Non-End-to-End Frameworks on COCO

On the COCO val2017 set, DualPose significantly surpasses bottom-up methods [10,42,43,44] and one-stage methods [11,13,27,45]. One-stage approaches like FCpose [11] and InsPose [45] with ResNet-50 [5] achieve even lower APs of 63.0% and 63.1%, respectively. Likewise, bottom-up methods like DEKR [42] and LOGO-CAP [44] obtain APs of 68.0 and 69.6, respectively. DualPose also outperforms previous top-down methods like PRTR [17] and shows comparable results with Poseur [46]. The results demonstrate that DualPose not only excels within top-down frameworks but also significantly outperforms all one-stage and bottom-up methods.

#### 4.2.3. Comparisons with End-to-End Frameworks on CrowdPose

We also conducted experiments on the CrowdPose [22] dataset to further validate the proposed model. Table 3 presents the performance metrics of leading competitors, including ED-Pose [30] and GroupPose [19]. The results indicate that our model achieves state-of-the-art performance, surpassing all other models across every evaluated metric. All experiments were conducted using 2 × NVIDIA A40 GPU, with Swin-L [23] as the backbone.

#### 4.2.4. Qualitative Results

**Figure 4:**
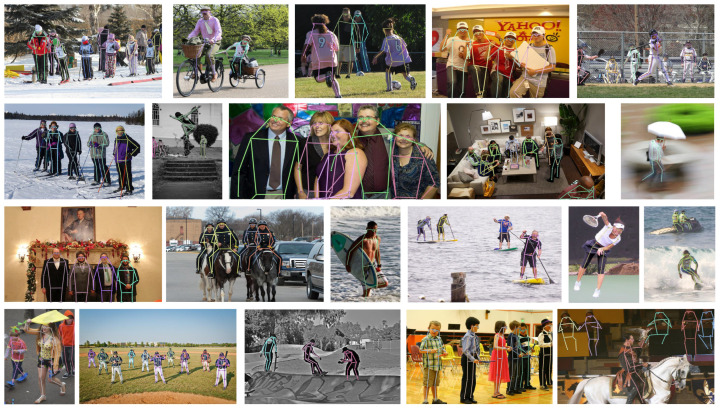
Visualization results of DualPose on MS COCO. An illustration of multi-person pose estimation in a busy environment. The model demonstrates its robustness in handling occlusions and densely populated scenarios by accurately detecting individuals and localizing keypoints.

Figure 4 presents the results obtained by DualPose, demonstrating its accuracy in multi-pose person estimation. Each image depicts a complex scenario, where the model successfully distinguishes between instances and accurately identifies key points. The colorful skeletons highlight the effectiveness of our approach, showing the model’s robustness in crowded or partially occluded scenes.

**Figure 5:**
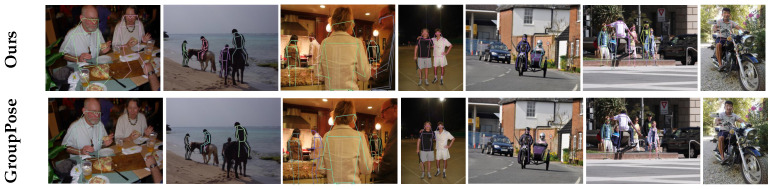
Qualitative analysis. Qualitative comparison between DualPose (top row) and GroupPose (bottom row). The superimposed postures show the differences in multi-person pose estimation performance, highlighting improvements in keypoint localization and pose consistency achieved by our method.

While DualPose achieves state-of-the-art performance, challenges remain in cases of severe occlusions, where overlapping keypoints may lead to misassignments (see Figure 5). Additionally, unusual poses or complex backgrounds can cause minor localization errors.

### 4.3. Ablations

We conducted a series of ablation experiments to assess the impact of each component and set each parameter of DualPose. Using ResNet-50 as the backbone, all results are reported on the COCO val2017 dataset with 60 epochs of training, unless otherwise noted.

**Query Design for Pose Estimation.** In the DualPose model, accurate pose estimation relies on both instance (inst) and keypoint (kpt) queries. As Table 4(a) illustrates, the experiments underscore the crucial role of keypoint queries. In this ablation study, the dual-block architecture remains unchanged, while adjustments are made to the input queries for the decoder. When only one type of query is used, the other is omitted, and the remaining queries handle both classification and keypoint regression. The model performs best when both instance and keypoint queries are present, with AP of 71.2%, AP_*M*_ of 66.2%, and AP_*L*_ of 79.0%. Performance decreases slightly when utilizing only keypoint queries (AP = 69.5%) and drops significantly when only instance queries are used (AP = 63.4%). These results clearly show that leveraging both query types is essential to achieve optimal results in human detection tasks.

**Number of Instance Queries.** As shown in Table 4(a), we also analyzed how different numbers of instance queries affect performance. The best results are obtained with 100 queries, and no further improvement is observed when that number is increased. For instance, using 200 queries results in a slight decrease (AP = 78.8%), while reducing queries to 50 lowers the AP to 70.7%. Consequently, 100 queries strike the best balance between computational efficiency and performance.

**Number of Denoising Queries.** The impact of the quantity of denoising queries on the model’s performance is examined in Table 4(c). The optimal balance is achieved with 50 queries as the default. Increasing the number of queries to 100 does not offer additional gains and slightly reduces AP_*L*_ to 78.8%, while dropping to 25 queries lowers the overall AP to 70.7%, AP_*M*_ to 65.8%, and AP_*L*_ to 78.5%. These findings imply that the extra computational cost is not justified when denoising queries exceed 50.

**Component Analysis.** Table 5 provides a comprehensive overview of each novel component in the proposed model. Incorporating the denoising mechanism improves AP by 0.3 points (from 70.6% to 70.9%). Adding the contrastive method further boosts the AP to 71.0%. Finally, introducing the split attention mechanism raises AP to a peak of 71.2%. All other metrics also improve consistently, demonstrating how each innovation enhances DualPose’s state-of-the-art performance.

**Dual-Block Design.** In DualPose, the class and keypoint blocks are essential, each focusing on classification and keypoint localization tasks, respectively. Each block is tested individually by “disabling” the other to observe the effect on performance. As shown in Table 6, the model achieves optimal performance when both Class-Block and Keypoint-Block are active. When using only the Keypoint-Block, there is a small decrease in performance (AP = 69.5%), whereas relying solely on the Class-Block leads to a more significant drop (AP = 68.1%). These findings indicate that both blocks are crucial for maximizing accuracy in multi-person pose estimation.

**Lambda Analysis.** Table 7 reports the ablation study on the two contrastive hyper-parameters used by CDN. Disabling the module entirely ($\lambda_{1}=\lambda_{2}=0$) causes a drop of −1.2 AP, confirming the importance of contrastive denoising. Keeping the default ratio but enlarging only the negative hyper-parameter ($\lambda_{1}=0.50,\lambda_{2}=2.00$) reduces performance by −0.3 AP, while doubling the positive hyper-parameter ($\lambda_{1}=1.00,\lambda_{2}=2.00$) yields a slightly larger decline −0.4 AP. The default choice ($\lambda_{1}=0.50,\lambda_{2}=2.00$) therefore offers the best trade-off and demonstrating that DualPose benefits from a narrow reconstruction band and a harder contrastive margin.

#### Inference Speed and FLOPs

Table 8 compares the performance of multi-person pose estimation methods (i.e., PETR [28], QueryPose [29], ED-Pose [30], GroupPose [19], and DualPose) across two different input resolutions (480 × 800 and 800 × 1333). FPS and inference time (ms) are computed on a platform equipped with an NVIDIA A40 GPU.

DualPose exhibits comparable computational performance to GroupPose, achieving similar frame rates while offering higher accuracy. Specifically, DualPose operates at 50.4 FPS (20 ms) at a resolution of 480 × 800 and 26.0 FPS (38 ms) at 800 × 1333, demonstrating an efficient balance between speed and accuracy.

With a ResNet-50 backbone, DualPose comprises 59,898,486 trainable parameters (around 59.9M) and requires 31.70 GFLOPs for a 480×800 input or 88.49 GFLOPs for an 800×1333 input.

## 5. Conclusions

In this paper, we present DualPose, a framework for multi-person pose estimation based on a dual-block transformer decoder architecture. The main contribution is related to the new transformer decoder architecture proposed, which decouples the computation of keypoints and class predictions into two distinct blocks, allowing each one to process the queries independently and more effectively. DualPose enhances accuracy and precision by implementing parallel group self-attentions and distinguishing class from keypoint predictions. Furthermore, training robustness and stability are improved through a contrastive denoising mechanism. These improvements make DualPose an excellent human pose estimation system applicable in all real cases, from industrial to surveillance applications, further enhancing the skills of smart sensors. Comprehensive experiments on MS COCO and CrowdPose demonstrate that DualPose outperforms state-of-the-art techniques.

## Figures and Tables

**Table 1:** Comparative evaluation using state-of-the-art for MS COCO val2017. Reference (Ref) values from previous frameworks are included in this section for context. The terms heatmap-based losses, human box regression losses, and keypoint regression losses are represented by the acronyms “HM”, “BR”, and “KR”, in that order. The term “RLE” denotes Poseur’s residual log-likelihood estimation. † denotes the flipping test. ‡ eliminates the Poseur prediction uncertainty estimation for an equitable regression comparison. (**Bold**: best. Underline: second best. * indicates retrained model on 2 × A40 GPU).

Method			Ref	Backbone	Loss	AP	AP_50_	AP_75_	AP_*M*_	AP_*L*_
**Non-End-to-End**	**Top-Down**	Mask R-CNN [5]	CVPR 17	ResNet-50	HM	65.5	87.2	71.1	61.3	73.4
		Mask R-CNN [5]	CVPR 17	ResNet-101	HM	66.1	87.4	72.0	61.5	74.4
		PRTR ^†^ [17]	CVPR 21	ResNet-50	KR	68.2	88.2	75.2	63.2	76.2
		Poseur ^‡^ [46]	ECCV 22	ResNet-50	RLE	70.0	-	-	-	-
		Poseur [46]	ECCV 22	ResNet-50	RLE	**74.2**	89.8	**81.3**	**71.1**	80.1
	**Bottom-Up**	HrHRNet ^†^ [10]	CVPR 20	HRNet-w32	HM	67.1	86.2	73.0	61.5	76.1
		DEKR ^†^ [42]	CVPR 21	HRNet-w32	HM	68.0	86.7	74.5	62.1	77.7
		SWAHR ^†^ [43]	CVPR 21	HRNet-w32	HM	68.9	87.8	74.9	63.0	77.4
		LOGO-CAP ^†^ [44]	CVPR 22	HRNet-w32	HM	69.6	87.5	75.9	64.1	78.0
	**One-Stage**	DirectPose [13]	-	ResNet-50	KR	63.1	85.6	68.8	57.7	71.3
		CenterNet ^†^ [27]	-	Hourglass-104	KR + HM	64.0	-	-	-	-
		FCpose [11]	CVPR 21	ResNet-50	KR + HM	63.0	85.9	68.9	59.1	70.3
		InsPose [45]	ACM MM 21	ResNet-50	KR + HM	63.1	86.2	68.5	58.5	70.1
**End-to-End**	**Previous Works**	PETR * [28]	CVPR 22	ResNet-50	HM + KR	67.4	86.1	74.9	61.2	76.0
		PETR * [28]	CVPR 22	Swin-L	HM + KR	71.6	89.3	79.3	65.8	79.9
		QueryPose * [29]	NeurIPS 22	ResNet-50	BR + RLE	68.0	86.9	72.9	62.2	75.0
		QueryPose * [29]	NeurIPS 22	Swin-L	BR + RLE	71.9	89.5	77.7	66.9	79.9
		ED-Pose * [30]	ICLR 23	ResNet-50	BR + KR	69.8	87.9	76.4	64.4	78.2
		ED-Pose * [30]	ICLR 23	Swin-L	BR + KR	72.0	89.9	79.9	67.1	81.2
		GroupPose * [19]	ICCV 23	ResNet-50	KR	70.4	88.2	77.8	65.1	78.3
		GroupPose * [19]	ICCV 23	Swin-L	KR	72.8	90.0	80.6	68.0	81.6
	**Ours**	DualPose	-	ResNet-50	KR	71.2	88.9	77.7	66.2	79.0
		DualPose	-	Swin-T	KR	72.2	89.4	78.7	67.6	80.4
		DualPose	-	Swin-L	KR	73.4	**91.8**	80.7	69.0	**82.3**

**Table 2:** Comparisons with state-of-the-art methods on MS COCO test-dev2017 dataset. Notations are consistent with Table 1.

Method			Ref	Backbone	Loss	AP	AP_50_	AP_75_	AP_*M*_	AP_*L*_
**Non-End-to-End**	**Top-Down**	Mask R-CNN [5]	CVPR 17	ResNet-50	HM	63.9	87.7	69.9	59.7	71.5
		Mask R-CNN [5]	CVPR 17	ResNet-101	HM	64.3	88.2	70.6	60.1	71.9
		PRTR ^†^ [17]	CVPR 21	ResNet-50	KR	68.8	89.9	76.9	64.7	75.8
		PRTR ^†^ [17]	CVPR 21	HRNet-w32	KR	71.7	90.6	79.6	67.6	78.4
	**Bottom-Up**	HrHRNet ^†^ [10]	CVPR 20	HRNet-w32	HM	66.4	87.5	72.8	61.2	74.2
		DEKR ^†^ [42]	CVPR 21	HRNet-w32	HM	67.3	87.9	74.1	61.5	76.1
		SWAHR ^†^ [43]	CVPR 21	HRNet-w32	HM	67.9	88.9	74.5	62.4	75.5
		LOGO-CAP ^†^ [44]	CVPR 22	HRNet-w32	HM	68.2	88.7	74.9	62.8	76.0
	**One-Stage**	DirectPose [13]	-	ResNet-50	KR	62.2	86.4	68.2	56.7	69.8
		CenterNet ^†^ [27]	-	Hourglass-104	KR + HM	63.0	86.8	69.6	58.9	70.4
		FCpose [11]	CVPR 21	ResNet-50	KR + HM	64.3	87.3	71.0	61.6	70.5
		InsPose [45]	ACM MM 21	ResNet-50	KR + HM	65.4	88.9	71.7	60.2	72.7
**End-to-End**	**Previous Works**	PETR * [28]	CVPR 22	ResNet-50	HM + KR	66.2	88.1	73.8	60.2	74.5
		PETR * [28]	CVPR 22	Swin-L	HM + KR	68.9	89.8	76.9	63.5	76.2
		QueryPose * [29]	NeurIPS 22	Swin-L	BR + RLE	70.4	90.6	77.0	65.7	77.7
		ED-Pose * [30]	ICLR 23	ResNet-50	BR + KR	68.1	88.9	75.8	62.9	75.8
		ED-Pose * [30]	ICLR 23	Swin-L	BR + KR	71.3	90.8	79.2	66.2	78.4
		GroupPose * [19]	ICCV 23	ResNet-50	KR	68.4	89.1	76.2	63.3	76.6
		GroupPose * [19]	ICCV 23	Swin-L	KR	71.5	90.9	79.6	66.3	79.3
	**Ours**	DualPose	-	ResNet-50	KR	69.5	89.4	76.6	64.4	77.4
		DualPose	-	Swin-T	KR	70.8	90.1	77.2	65.3	78.5
		DualPose	-	Swin-L	KR	**71.8**	**92.2**	**79.8**	**67.9**	**80.1**

**Table 3:** Comparisons with state-of-the-art methods on CrowdPose test dataset. Notations are consistent with Table 1. Swin-L is used as the backbone. * indicates retrained model on 2 × A40 GPU.

Method	Loss	AP	AP_50_	AP_75_	AP_*E*_	AP_*M*_	AP_*H*_
PETR * [28]	HM+KR	70.0	88.7	77.0	75.8	70.5	64.5
QueryPose * [29]	BR+RLE	71.1	90.2	76.6	77.8	71.9	63.8
ED-Pose * [30]	BR+KR	71.5	88.7	78.1	79.0	72.1	62.3
GroupPose * [19]	KR	72.7	90.0	79.0	79.5	73.2	64.8
DualPose	KR	**72.9**	**90.8**	**79.2**	**81.3**	**74.5**	**66.1**

**Table 4:** Ablation experiments forDualPose’s queries evaluated on MS COCO val2017. Default settings are marked in gray. (a) Designing queries for pose estimation. Both instance (inst) and keypoint (kpt) queries are crucial, with a particular emphasis on the importance of keypoint queries. (b) Number of instance queries. Testing indicates that setting the number of queries to 100 results in better performance compared to using either 50 or 200 queries. (c) Number of denoising queries. The default setting is 50 queries. Exceeding 50 queries results in similar performance but with longer execution times.

q. Types	AP	AP_*M*_	AP_*L*_	#IQ	AP	AP_*M*_	AP_*L*_	#DQ	AP	AP_*M*_	AP_*L*_
inst & kpt	**71.2**	**66.2**	**79.0**	50	70.1	65.2	77.3	25	70.7	65.8	78.5
only kpt	69.5	64.0	77.9	100	**71.2**	**66.2**	**79.0**	50	**71.2**	**66.2**	**79.0**
only inst	63.4	61.5	70.0	200	70.9	**66.2**	78.8	100	71.0	**66.2**	78.8
(a)				(b)				(c)			

**Table 5:** Component analysis. Performance improvements obtained with the method components.

Model	AP	AP_50_	AP_75_	AP_*M*_	AP_*L*_
Dual-Block	70.6	88.5	77.1	65.5	78.3
+Denoising	70.9	88.7	77.5	65.8	78.5
+Contrastive	71.0	88.8	77.5	**66.2**	**79.0**
+Split	**71.2**	**88.9**	**77.7**	**66.2**	**79.0**

**Table 6:** Dual-block design. Both class and keypoint block are essential to achieve the best performance.

Configuration	AP	AP_*M*_	AP_*L*_
DualPose (Class-Block + Keypoint-Block)	**71.2**	**66.2**	**79.0**
Class-Block Only (Keypoint-Block disabled)	64.7	62.0	70.4
Keypoint-Block Only (Class-Block disabled)	69.9	64.9	78.3

**Table 7:** Sensitivity of CDN to the positive ($\lambda_{1}$) and negative ($\lambda_{2}$) noise hyper-parameters.

$\lambda_{1}$	$\lambda_{2}$	AP	AP_50_	AP_75_	AP_*M*_	AP_*L*_
0	0	70.0	87.5	76.5	65.4	76.7
0.50	1.50	**71.2**	**88.9**	**77.7**	**66.2**	**79.0**
0.50	2.00	70.9	88.6	77.3	66.0	78.8
1.00	1.50	70.8	88.4	77.0	65.8	78.2

**Table 8:** Comparison of FPS and time (ms) for different methods at various input resolutions with ResNet-50 on a NVIDIA A40 GPU. (**Bold**: best. Underline: second best).

Method	480 × 800 (FPS ↑/Time ↓)	800 × 1333 (FPS ↑/Time ↓)
PETR [28]	18.0/55 ms	10.2/98 ms
QueryPose [29]	16.5/61 ms	11.7/85 ms
ED-Pose [30]	33.3/30 ms	21.2/47 ms
GroupPose [19]	**52.6/19 ms**	**27.7/36 ms**
DualPose	50.4/20 ms	26.0/38 ms

## Data Availability

The source code together with the pretrained weights are published at the following link: https://github.com/iot-unimore/DualPose (accessed on 1 May 2025). The datasets used for training and testing the networks are publicly available (see references in the paper).

## Abbreviations

The following abbreviations are used in this manuscript:

CDN	contrastive denoising
OKS	object keypoint similarity
HM	heatmap-based losses
BR	human box regression losses
KR	keypoint regression losses
RLE	Poseur’s residual log-likelihood
AP	average precision
AP_*E*_, AP_*M*_, AP_*H*_	AP on easy/medium/hard crowding scenarios
AP_*M*_, AP_*L*_	AP on people of medium/large size
